# Automatic Measurement of Endometrial Thickness From Transvaginal Ultrasound Images

**DOI:** 10.3389/fbioe.2022.853845

**Published:** 2022-03-29

**Authors:** Yiyang Liu, Qin Zhou, Boyuan Peng, Jingjing Jiang, Li Fang, Weihao Weng, Wenwen Wang, Shixuan Wang, Xin Zhu

**Affiliations:** ^1^ Biomedical Information Engineering Lab, The University of Aizu, Aizuwakamatsu, Japan; ^2^ Department of Obstetrics and Gynecology, Tongji Hospital, Huazhong University of Science and Technology, Wuhan, China

**Keywords:** endometrial thickness, semantic segmentation, deep learning, transvaginal ultrasonography (TVUS), two-step method

## Abstract

**Purpose:** Endometrial thickness is one of the most important indicators in endometrial disease screening and diagnosis. Herein, we propose a method for automated measurement of endometrial thickness from transvaginal ultrasound images.

**Methods:** Accurate automated measurement of endometrial thickness relies on endometrium segmentation from transvaginal ultrasound images that usually have ambiguous boundaries and heterogeneous textures. Therefore, a two-step method was developed for automated measurement of endometrial thickness. First, a semantic segmentation method was developed based on deep learning, to segment the endometrium from 2D transvaginal ultrasound images. Second, we estimated endometrial thickness from the segmented results, using a largest inscribed circle searching method. Overall, 8,119 images (size: 852 × 1136 pixels) from 467 cases were used to train and validate the proposed method.

**Results:** We achieved an average Dice coefficient of 0.82 for endometrium segmentation using a validation dataset of 1,059 images from 71 cases. With validation using 3,210 images from 214 cases, 89.3% of endometrial thickness errors were within the clinically accepted range of ±2 mm.

**Conclusion:** Endometrial thickness can be automatically and accurately estimated from transvaginal ultrasound images for clinical screening and diagnosis.

## 1 Introduction

The endometrium is an epithelial tissue layer within a mammalian uterus, the physiology of which manifests differently across phases of the menstrual cycle (e.g., proliferative, secretory, menstrual). Uterine space-occupying lesions primarily include polyps, submucosal fibroids, endometrial hyperplasia, and endometrial adenocarcinoma Nalaboff et al. (2001). The prevalence of endometrial hyperplasia increases with age, with an overall estimate of 133/100,000 woman-years. It is rare in women under age 30 years and peaks in women aged 50–54.1 years Auclair et al. (2019). Endometrial polyps are common in women over age 35 years and their incidence increases with age. Previous publications have reported that the incidence of endometrial polyps is about 3% in women under age 35 years, about 23% in those over age 35 years, and is highest among postmenopausal women: 31% with the peak at age 50 years. Nevertheless, among infertile patients the prevalence of endometrial polyps has increased significantly, though the exact incidence is difficult to determine Huang and Xiang (2014).

Normal endometrium is uniform in thickness, homogeneous in echotexture, and has no submucosal or myometrial abnormality Davis et al. (2002). Endometrial thickness (ET) < 5 mm usually has a high negative predictive rate for endometrial disorders Berridge and Winter (2004). ET is considered abnormal when it exceeds 8 mm in the proliferative phase or 16 mm in the secretory phase Jorizzo et al. (1999). In premenopausal women, normal ET changes continuously across the menstrual cycle. In postmenopausal women, ET is among the most important indicators for endometrial malignancy risk screening. Though studies have shown different ET cutoff values, it is generally understood that risk for endometrial cancer is higher when ET ≥ 3–5 mm. However, endometrial diseases cannot be diagnosed by ET alone, and further examinations are required for ET values in this range Gupta et al. (2002).

Transvaginal ultrasonography (TVUS) the first-line diagnostic tool for identifying uterine cavity lesions Kolhe (2018), is also low-cost and the most convenient tool for detection and diagnosis of uterine lesions Turkgeldi et al. (2015). It produces high-resolution images of the uterus and endometrium because of the proximity of vaginal transducers to the uterus Wikland et al. (1992). Use of TVUS for uterine imaging provides useful information, like uterine and endometrial length and thickness, endometrial texture, and uterine position (e.g., flexion, versions) Park et al. (2019), allowing identification of abnormalities. TVUS is also recommended for screening endometrium-related diseases among postmenopausal women Shokouhi (2015).

Routine ultrasound-based diagnosis relies on manual operation and sonographer visual interpretation. In conventional ultrasound-based endometrium assessment, doctors measure a standard longitudinal section of the uterus using TVUS, manually measure ET, and simultaneously check for other abnormal conditions. Therefore, current diagnoses mainly rely on physician experience, possibly leading to significant interindividual measurement differences. This procedure is also time- and labor-intensive Ahuja (2019). Therefore, automated measurement may provide more consistent ET values and, therefore, facilitate diagnosis.

Recent studies have assessed computer-aided measurement of endometrium identity. For example, Hu et al. proposed a deep learning-based ET measurement using healthy participants’ TVUS images. Through validation using 277 images from 27 cases, their method resulted in an average Dice coefficient of 0.83. For thickness measurement, they achieved a mean absolute error (MAE) of 1.23/1.38 mm and a root mean squared error of 1.79/1.85 mm with different test sets Jorizzo et al. (1999). Park et al. introduced semiautomated endometrium segmentation from TVUS images using key point discriminators. Compared with traditional segmentation networks, their key-point segmentation method improved the performance of endometrium segmentation. Their average Dice coefficient and Jaccard coefficients were 82.67 and 70.46%, respectively Park et al. (2019). Ni et al. proposed a novel active contour-based segmentation method to segment uterine fibroids in a TVUS image sequence. Their method demonstrated low-level properties of shape matrices, greatly improving performance and robustness of active contouringNi et al. (2016). Quan et al. proposed a normalized cutoff method to segment tumor ultrasound image by simple linear iterative clustering; their method’s advantages are removing the effects of noise and weak edges in ultrasound images Quan et al. (2013).

Compared with traditional machine learning, deep learning can automatically learn high-level features in data and, therefore, reduce the complexity of manual feature design. Recently, deep learning has been applied not only to image segmentation, but to speech recognition, image recognition, and defect detection Hao et al. (2021) and Duan et al. (2021). Sun et al. developed a computer-aided diagnosis for endometrial diseases by histopathological images using a convolutional neural network and attention mechanisms Sun et al. (2019). Singhal et al. employed deep learned snake detection to assess endometrium using TVUS. They propose a hybrid variational curve propagation model which embeds a deep-learned endometrium probability map in the segmentation energy functional. In a database of 59 TVUS images, this solution improved performance by about 30% over contemporary supervised learning methods, and thickness measurements were found to be within ±2 mm of manual measurementsSinghal et al. (2017).

Herein, we developed a two-step method to automatically measure ET for the screening and diagnosis of endometrial diseases. First, we used semantic segmentation based on deep learning to segment endometria from 2D TVUS images. Then, we calculated the thickness of the endometrium based on the largest inscribed circle searching method. The method combines endometrium segmentation and automated ET measurement, and may be implemented in screening for, detecting, and diagnosing endometrial disorders.

## 2 Materials and Methods

### 2.1 Training and Validation Data

**FIGURE 1:**
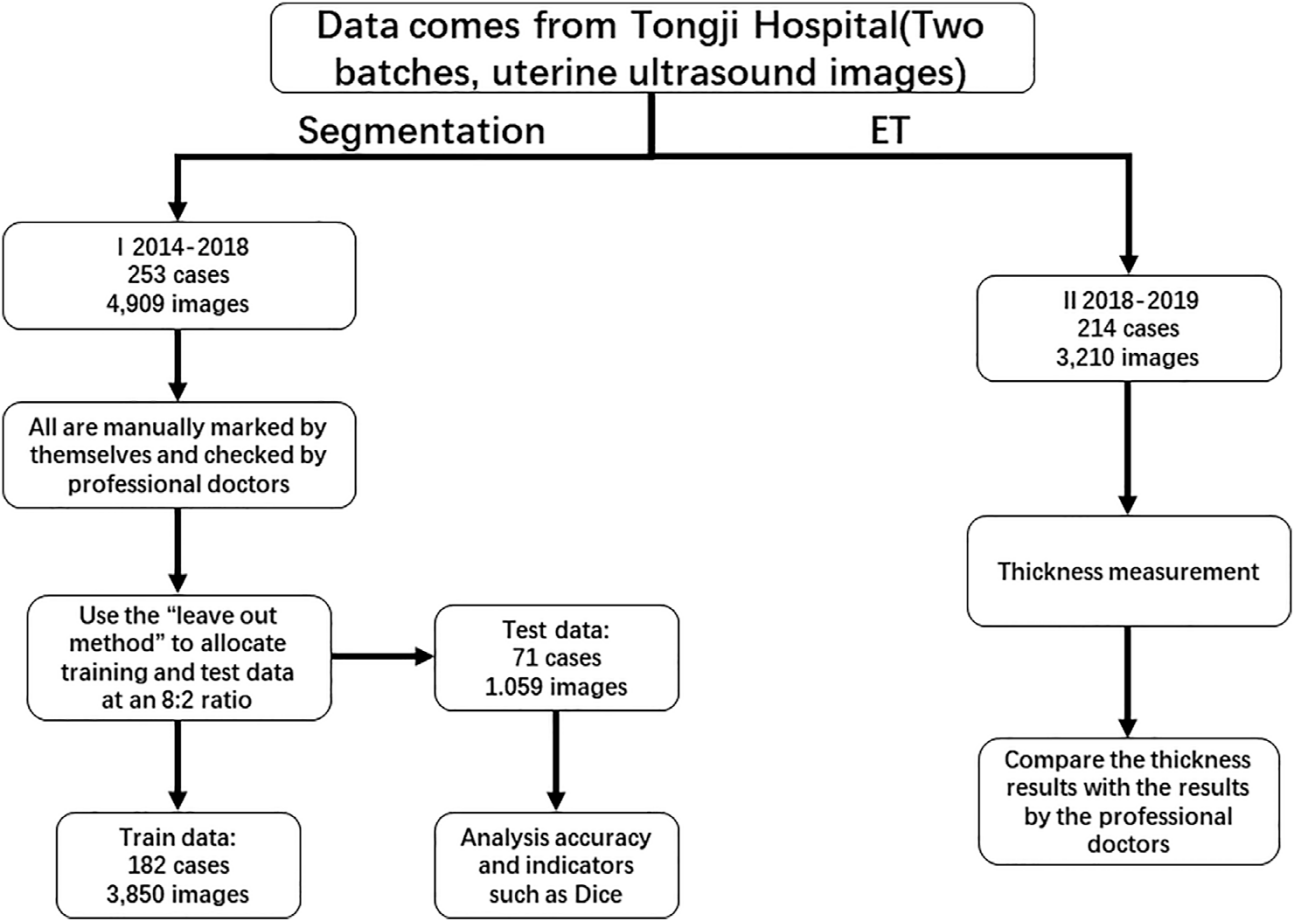
Study overview.

We collected 467 consecutive cases (ages 16–80 years) from 2014 to 2019 at the Gynecology and Obstetrics Division, Tongji Hospital, Huazhong University of Science and Technology. The TUVS devices were all GE Voluson E1 (GE Corp., United States). Two separate datasets were used for endometrium segmentation and ET measurement validation, respectively. For the first dataset, we employed the leave-out method to divide the dataset into training and validation data for the proposed endometrium segmentation method. We randomly selected 80% of cases for training; the remaining 20% were used for validation Allibhai (2018). As illustrated in Figure 1, the data for training and validation of segmentation included 4,909 images from 253 cases. We used 2,850 images from 182 cases for training and 1,059 images from 71 cases for validation. The other dataset contained approximately 3,210 images from 214 cases and was used to validate the ET measurement method. In that dataset, ET was measured manually by two professional sonographers (QZ and JJJ) as the gold standard.

**FIGURE 2:**
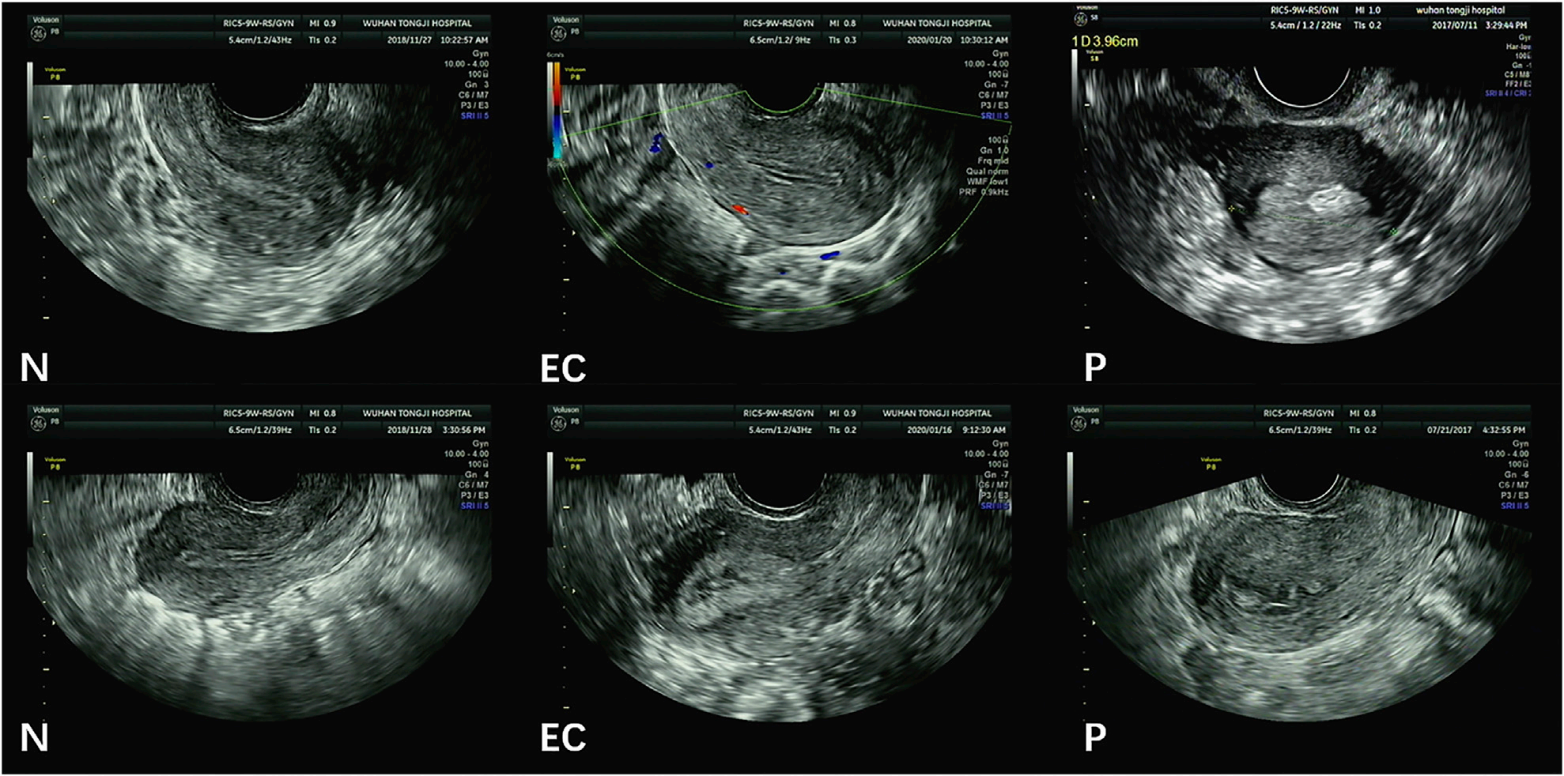
Sample TVUS images. (N = normal endometrium, EC = endometrial cancer, P = endometrial polyps).

Figure 2 illustrates image samples from patients with normal endometria, endometrial cancer, and endometrial polyps. All images were saved in DICOM, and their corresponding sizes, resolutions, and other information were in file headers. All images had a size of 852 × 1136 pixels.

This retrospective study was approved by the Institutional Review Board of Tongji Hospital, Huazhong University of Science and Technology.

### 2.2 Methods

#### 2.2.1 Endometrium Segmentation

**FIGURE 3:**
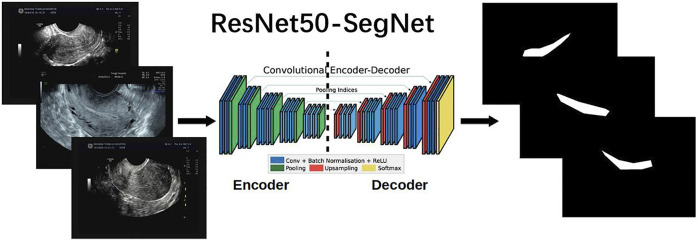
ResNet50-SegNet architecture [Modified from Badrinarayanan et al. (2015)] for semantic segmentation of endometrium from uterine ultrasound images.

We compared four state-of-the-art semantic segmentation algorithms through trial and error. Based on these comparisons, the best result was achieved by the SegNet-ResNet50 model. These details are described in the second part of the Supplementary Material. SegNet, a fully convolutional network for pixel-level image segmentation, was used to perform endometrium segmentations as illustrated in Figure 3. The core segmentation component was an encoder network and its corresponding decoder network, followed by a pixel-level classification network. SegNet can greatly reduce the number of training parameters in the encoder layer Badrinarayanan et al. (2017); Farabet et al. (2012). The coding network consists of 13 convolutional layers.

ResNet50 serves as the backbone of SegNet, and is used to extract features in images for segmentation Hu et al. (2019). ResNet50 has 50 layers and was pretrained using the ImageNet Large Scale Visual Recognition Challenge 2012 classification dataset, consisting of 1.2 million training images, with 1,000 classes of objects He et al. (2016). The residual block in ResNet is used to overcome network performance degradation caused by gradient dispersion in deep neural networks Hao et al. (2021). ResNet50 was pretrained using the ImageNet dataset composed of approximately 1 million images Deng et al. (2009).

The SegNet encoder is based on ResNet50, whose four convolution blocks are used, and each convolution module has four convolution layers. All the layers use rectified linear units (ReLU) as the classification function in a deep neural network Agarap (2018)), including 12 identity blocks each with three convolution layers. An average pooling (7 × 7) is used before the encoder output. The SegNet decoder includes five 3 × 3 convolutional layers.

ResNet50-SegNet and the corresponding programs were developed using the Tensorflow–Keras frameworksGéron (2019) and a workstation with GeForce RTX 3080 10 GB.

#### 2.2.2 Automated Measurement of ET

**FIGURE 4:**
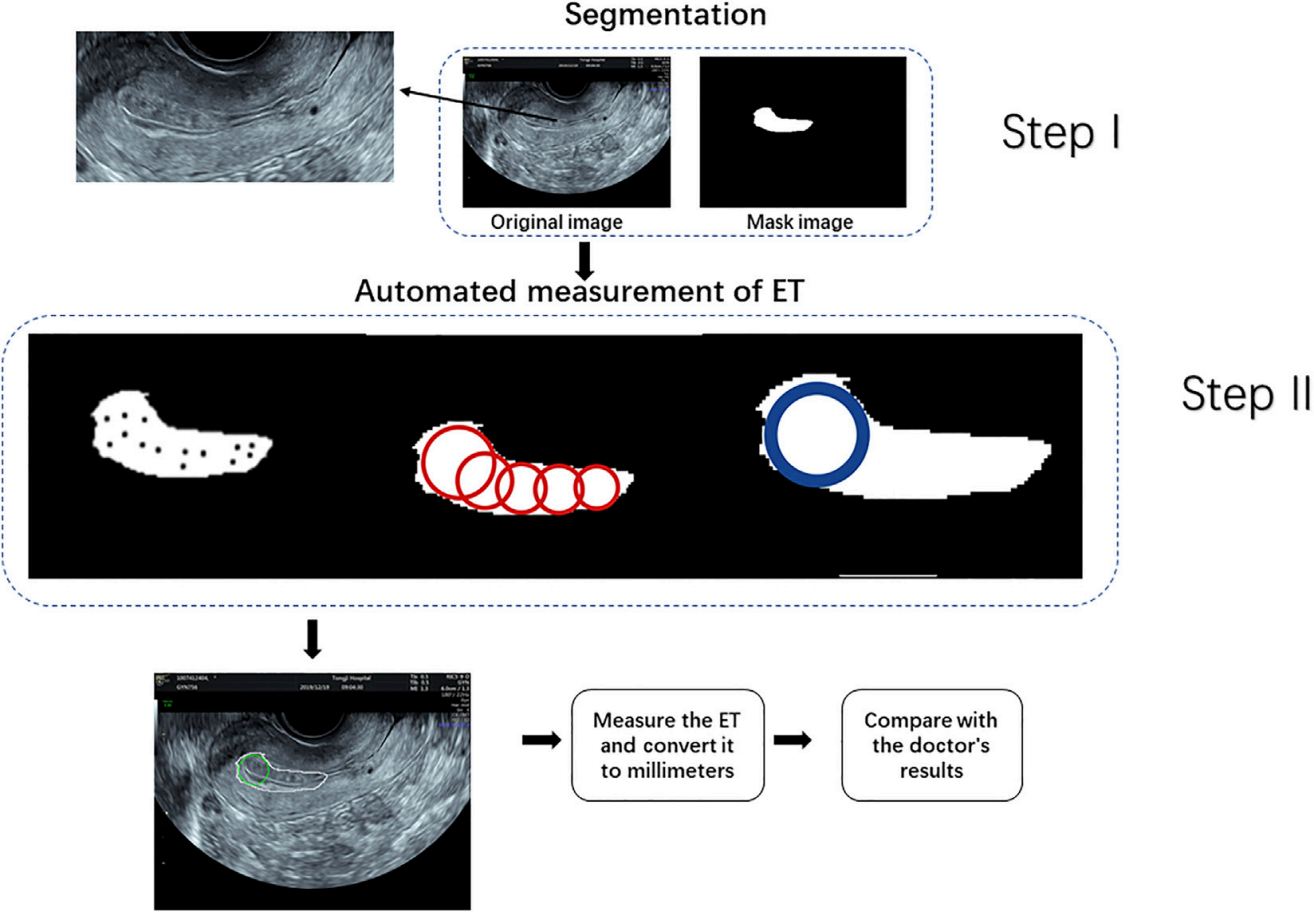
Procedure for measuring endometrial thickness.

**FIGURE 5:**
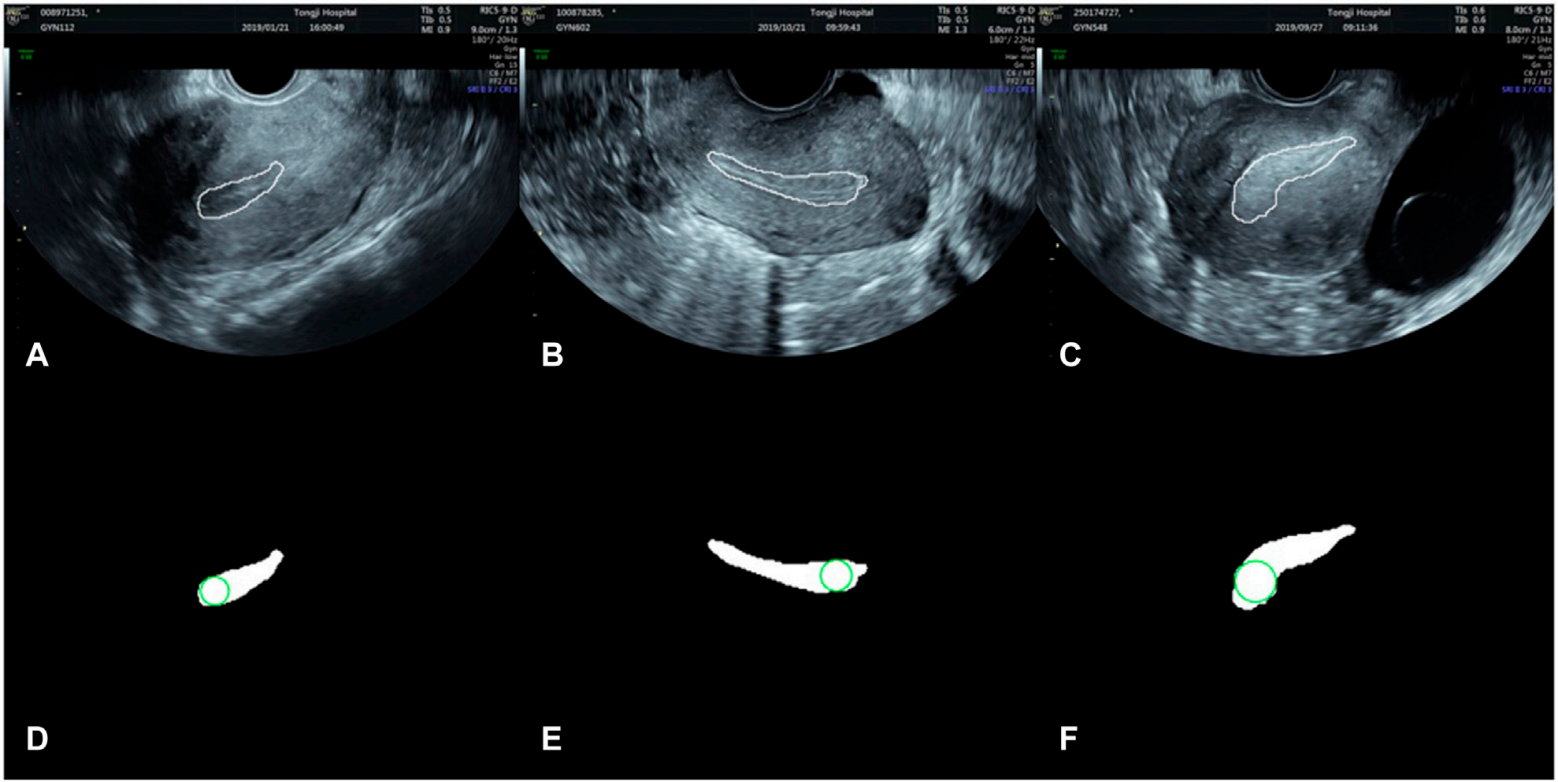
Automatically search the largest inscribed circle on the segmented endometrial mask. **(A–C)** are the segmentation results in the original images, and **(D–F)** the largest inscribed circles on the segmented endometrial masks.

After endometrium segmentation, we proposed what we call a ‘largest inscribed circle searching method’ to automatically measure ET. This exhaustive method is described in Figure 4. First, the largest connected region is searched from the endometrium segmentation results. The corresponding largest inscribed circle is then found through setting each pixel point in the region as the center of the circle. Figure 5 shows examples of ET results. The gold standard upon which automated measurements were based was ETs of all cases as measured by two professional sonographers (QZ and JJJ).

### 2.3 Evaluation Measures

The Dice coefficient measures the accuracy of image segmentation Jiang et al. (2021). Dice was defined as follows to serve as an index of similarity of measure between two samples (with values in the range [0, 1]) Shamir et al. (2019).

$$Dice\;coefficient=\frac{2\times TP}{2\times TP+FP+FN}$$ (1)

TP (true positive) indicates the number of correctly segmented pixels of endometrium, FP (false positive) the number of missed pixels of endometrium, and FN (false negative) the number of wrongly segmented pixels of non-endometrial regions Hao et al. (2021).

Mean absolute error (MAE) defined in Eq. 2 is used to quantify measurement errors in the automated ET measurement Willmott and Matsuura (2005).

$$MAE=\frac{\sum_{i=1}^{n}\left|y_{i}-x_{i}\right|}{n}=\frac{\sum_{i=1}^{n}\left|e_{i}\right|}{n}$$ (2)

where *y* is the ET measured by the proposed method, *x* is the gold standard (herein measures by the two professional gynecologists), *e* is the absolute error, and *n* is the total number of validation images. The smaller MAE is, the better the accuracy of the automated ET measurement. We also defined an acceptable rate for the measurement of ET as follows:

$$Acceptable\;rate=\frac{\sum_{i=1}^{n}\left|y_{i}-x_{i}\right|<2\text{mm}}{n}.$$ (3)

## 3 Results

### 3.1 Endometrium Segmentation

**FIGURE 6:**
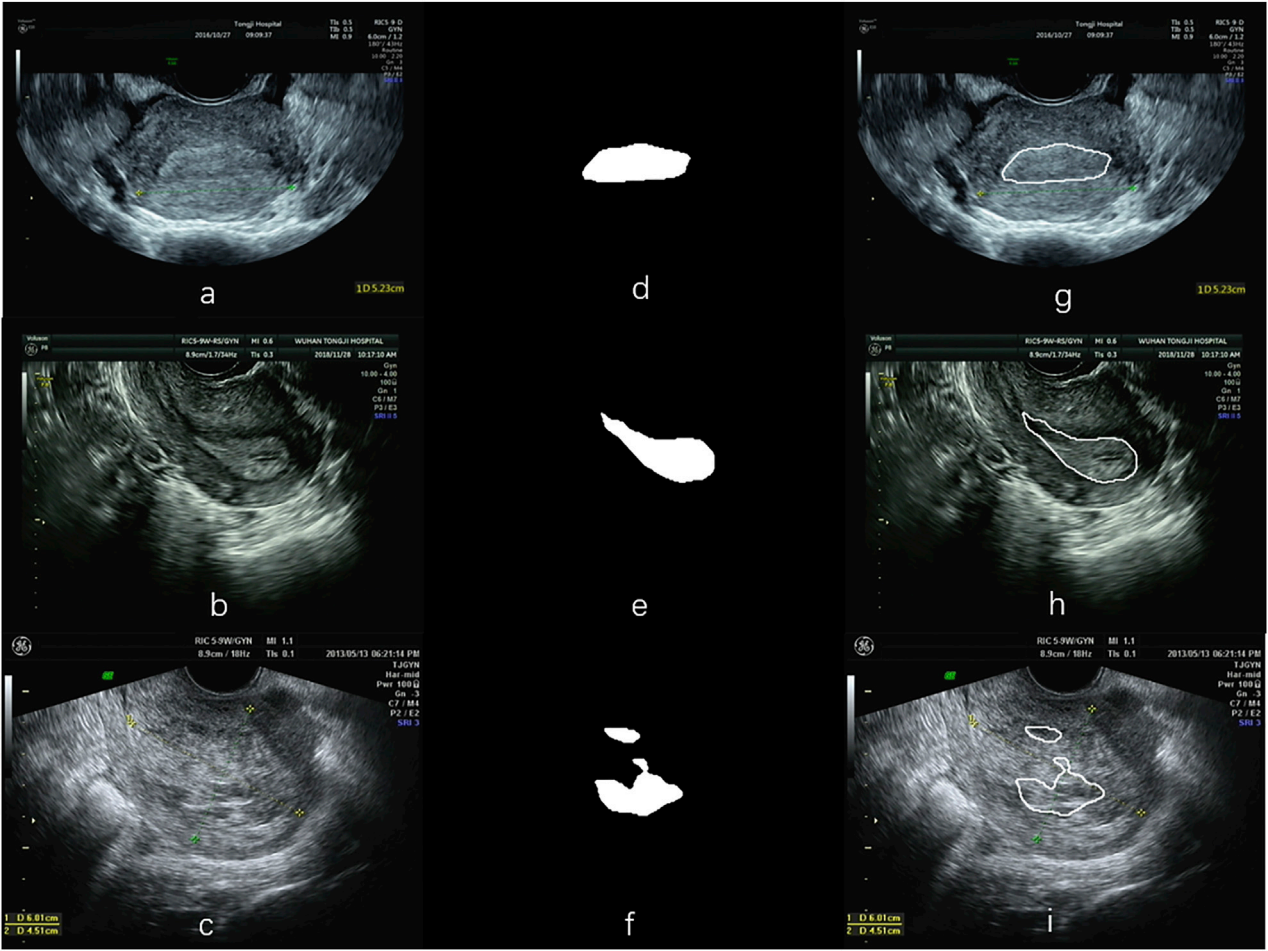
Examples of segmentation using the ResNet50-SegNet model. **(A–C)** are the original images, **(D–F)** the segmented endometrial masks, and **(G–I)** the segmentation results in the original images.

**FIGURE 7:**
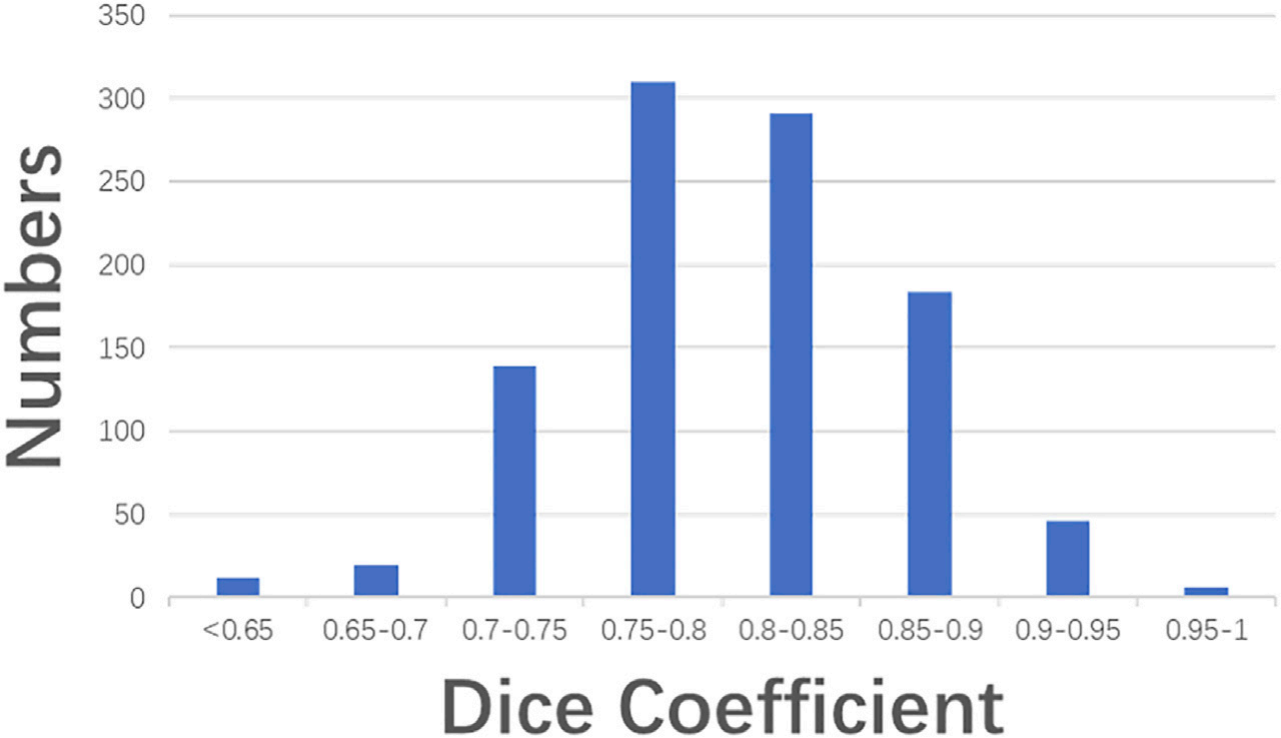
Histogram of Dice coefficient in the validation images.

Figure 6 illustrates three segmentation examples obtained by the Resnet50-SegNet model. Figures 6A–C,D–F,G–I show original images, ground truth, and segmentation results, respectively. The Dice coefficient for the segmentation results in Figures 6G–I were 0.93, 0.74, and 0.38, respectively. Figure 7 shows the histogram of Dice coefficient in the validation images. The average Dice coefficients and its standard deviation were 0.81 and 0.52, respectively.

### 3.2 ET Measurement Errors

**FIGURE 8:**
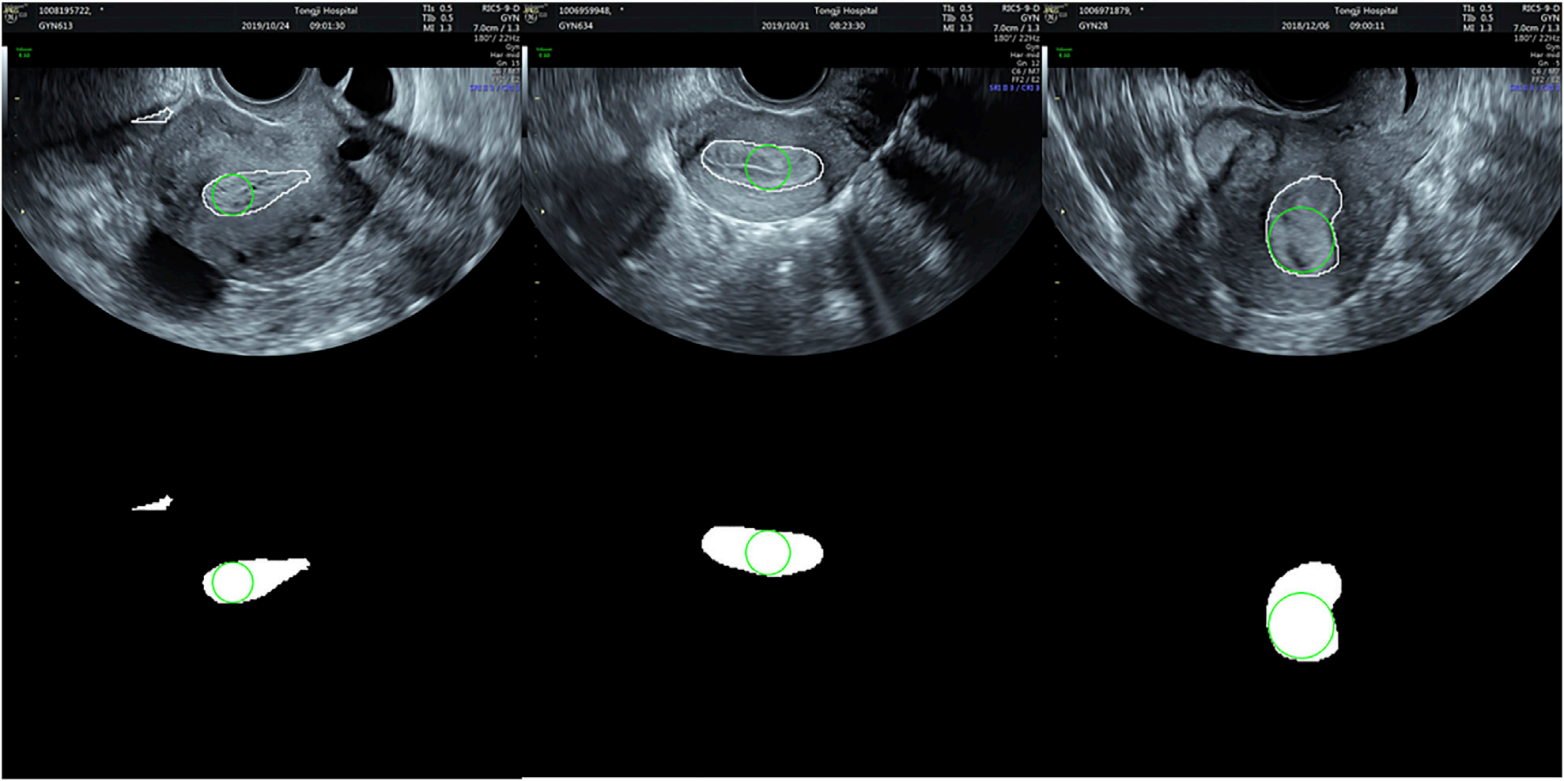
The largest inscribed circles in three segmented mask images.

**FIGURE 9:**
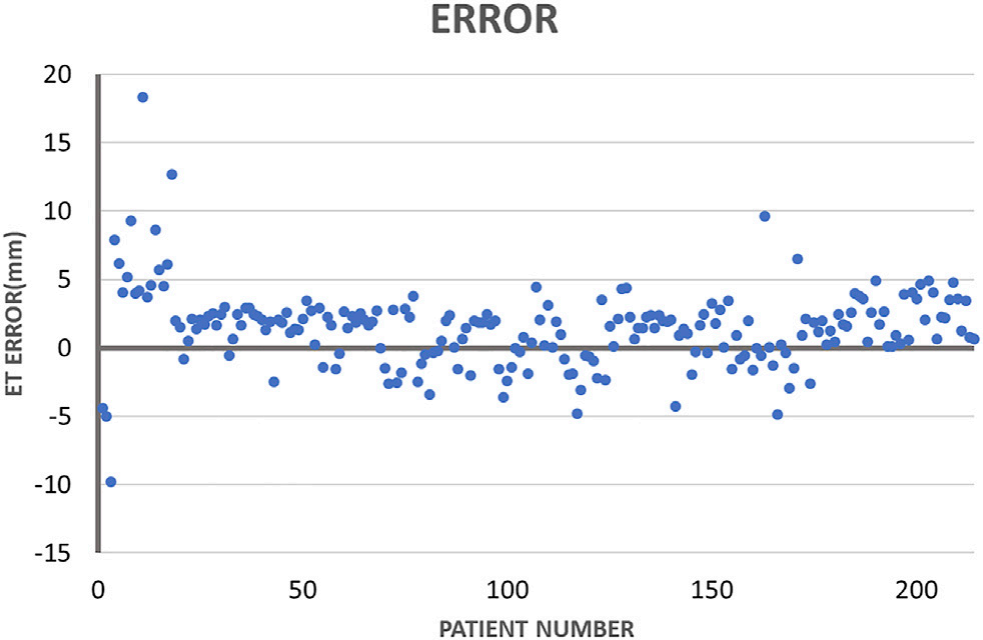
Absolute endometrial thickness errors measured by the proposed method.

Figure 8 illustrates the largest inscribed circles in three segmented mask images. Figure 9 shows the ET errors for 214 cases in the validation dataset. In 191 cases, ET errors were within the range ±3 mm, i.e., the acceptable margin of error. Thus, the acceptable rate was 89.3%. Table 1 lists the statistical results of ET measurement in three categories with ET ≤ 3 mm, 3\lt ET ≤ 10 mm and ET > 10 mm. The proposed uterine endometrium measurement system showed good performance for cases with ET > 3 mm. The acceptable rate in the 3 < ET ≤ 10 mm and ET > 10 mm group reached 95.4 and 98.3%, respectively. For cases with ET ≤ 3 mm and ET > 3 mm, the MAE was 3.6 and 2.0 mm, respectively.The MAE for all validation data was 2.3 mm.

**TABLE 1:** Measurement results

Classification	Number of cases	Number of cases with errors within ±3 mm	Number of cases with errors over ±3 mm	Acceptable rate (%)
ET ≤ 3 mm	22	12	10	54.5
3 mm < ET ≤ 10 mm	130	124	6	95.4
ET > 10 mm	62	61	1	98.3
Total	214	191	23	89.3

To confirm the influence of pathology on performance, we also divided the 214 cases into three categories (N, P, EC) according to pathology, using t-tests to confirm whether there were significant differences in ET errors among them.

Tables 2−4 list the statistics and average errors in the three categories. Table 2 shows result for the accurate segmentation results; Tables 3, 4 show results for the inaccurate segmentation results. Data with inaccurate segmentation results would be divided into two cases. First, when ET ≤ 3 mm, the inner membrane appears as a very thin, bright line; thus, 22 cases were difficult to segment. Second, the boundaries of eight case images were unclear, making it impossible to accurately identify the position of the endometrium.

**TABLE 2:** Average ET measurement errors in cases with errors ≤ 2 mm

Type	Case number	Avg error (mm)
P	47	1.28
EC	10	1.21
N	127	1.16
Total	184	1.22

**TABLE 3:** Average errors of ET in cases with ET > 3 mm and errors > 2 mm

Type	Case number	Avg error (mm)
P	12	4.63
EC	2	5.82
N	8	3.94
Total	22	4.58

**TABLE 4:** Average errors of ET in cases with ET ≤ 3 mm and errors > 2 mm

Type	Case number	Avg error(mm)
P	0	−
EC	0	−
N	8	4.46
Total	8	4.46

Table 5 shows that the error values of these results conform to the normal distribution. Furthermore, t-tests showed no significant differences between the three data categories.

**TABLE 5:** Measurement results within diseases categories

	N	P	EC	N *vs*. P	P *vs.* EC	N *vs*. EC
Mean	1.32	0.87	1.38
Variance	10.62	5.99	5.06			
Observations	127	43	10			
P(T ≤ t) one-tail	0.17				0.27	0.47
P(T ≤ t) two-tail	0.34				0.54	0.94
*P(Corrected by Bonferroni) one-tail	0.30			
*P(Corrected by Bonferroni) two-tail	0.60			

## 4 Discussion

The endometrium is the innermost glandular layer, forming the inner lining of the uterus, which thickens and sheds cyclically. Many endometrial studies have been performed on asymptomatic or postmenopausal women. Janesh et al. suggested that ET of ≤5 mm can exclude endometrial pathology and eliminate the need for endometrial sampling for histologic examinations Gupta et al. (2002). However, endometrial-related diseases are now occurring more frequently among younger women. According to an official report by the World Health Organization, women globally, from all ethnic groups and social classes, suffer from endometrial diseases; onset can occur any time from first menstruation (menarche) to menopause World Health Organization (2021). Therefore, screening for endometrial diseases is equally important for menstrual and menopausal women.

Ultrasound-based diagnosis of endometrial diseases has shown irreplaceable superiority, greatly reducing patient economic burden. Since measurement by different sonographers can lead to inconsistent results, an objective and automated measurement method would be preferable.

Our endometrium segmentation results show that the proposed method provides more accurate segmentation compared with conventional networks. This is consistent with previous results by Park et al. Park et al. (2019). However, that group proposed a semiautomated endometrium segmentation from TVUS images using key point discriminators. Hu et al. proposed a deep learning-based thickness measurement from TVUS images from healthy participants Hu et al. (2019). In contrast, our study was not limited to those with normal, healthy endometria, but included cases with endometrial cancer and polyps, in whom endometrium segmentation is challenging because the endometrium is usually irregular and difficult to identify. Regarding segmentation methods, there is no universal medical image segmentation method due to the complicated nature of various medical images Li et al. (2019). Therefore, the choice of segmentation method might be task-specific. We have compared several state-of-the-art semantic segmentation algorithms through trial and error. Through this process, ResNet50-SegNet demonstrated the best performance in the segmentation of endometrium in TVUS. Bhatnagar et al. also found that ResNet50-SegNet was the best method for UAV image segmentation related to identifying raised swamp vegetation types Bhatnagar et al. (2020).

The ET measurement results from the method developed herein were compared with gold standard manual ET measurements. The novel method showed a high accuracy for measuring ET, especially in cases with ET > 3 mm. Furthermore, there was no difference in the accuracy of this ET detection among the three endometrial disorder types. Therefore, this system may help doctors complete this assessment more quickly and accurately.

Although this method shows good performance for ET measurement, the study has some limitations. First, data regarding endometria <3 mm cannot be guaranteed as correct, as the accuracy rate was only 55.3%. Because of the measurement error of the inner membrane less than 3mm, false positive results may result, leading to patients undergoing further invasive examinations, such as diagnosing curettage or hysteroscopy. Therefore, we will, in our future research, continue to adjust and optimize the segmentation model to solve this problem. Second, the doctors involved in measuring ET herein worked at the same hospital and used GE Voluson E1. Thus, inter-hospital difference could not be considered. Third, identifying malignancy was based not only on ET, but on age at the time of the test, menstrual cycle, pregnancy status, and whether hormone therapy was used Liu and Chang (2004).

Video images herein were currently treated as independent, static frames. In the future, we will explore deep learning algorithms, like recurrent neural networks, to exploit the temporal information among frames. The endometrium might be better segmented when assessed using adjacent frames. Use of other factors such as participant age, uterine orientation, and surgical history might also improve ET measurement.

## 5 Conclusion

We have presented an automated ET measurement system using TVUS images. Using a ResNet50-based SegNet, we achieved a Dice coefficient of 0.82 for the segmentation test set. Using the largest inscribed circle searching method, ET was obtained from the segmentation results for satisfactory measurement accuracy. Validation based on images from 214 participants showed that 89.3% of measurement errors were within the clinically acceptable range, i.e., ≤2 mm. The proposed method may markedly improve both efficiency and efficacy of ultrasonic endometrium diagnosis.

## Data Availability

The datasets presented in this article are not readily available because the restrictions of IRB. Requests to access the datasets should be directed to WW, petrawang@163.com.
