# Supplementary figures and images for: Automatic Measurement of Endometrial Thickness From Transvaginal Ultrasound Images
Source: Front Bioeng Biotechnol. 2022 Mar 29;10:853845. doi: 10.3389/fbioe.2022.853845 (PMC9001908; doi:10.3389/fbioe.2022.853845)

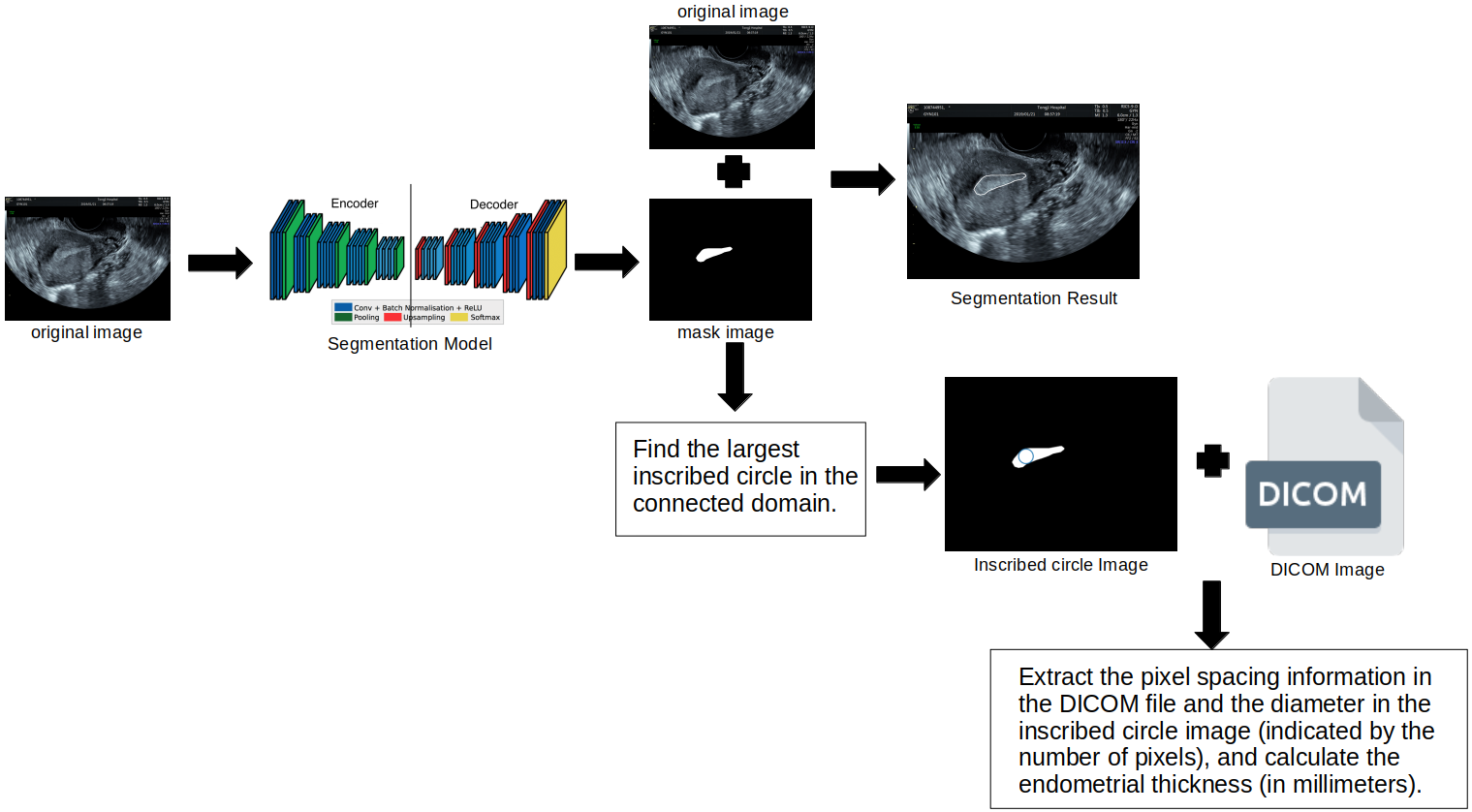

Supplement: Supplementary file 1 [file Image1.TIFF]

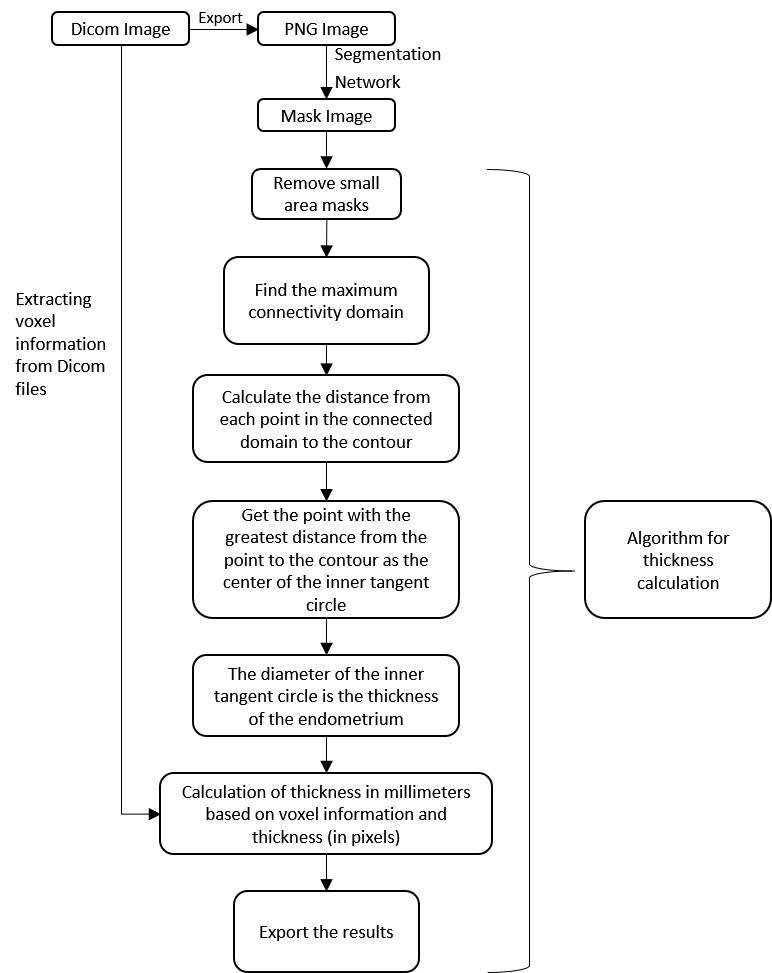

Supplement: Supplementary file 2 [file Image3.TIF]

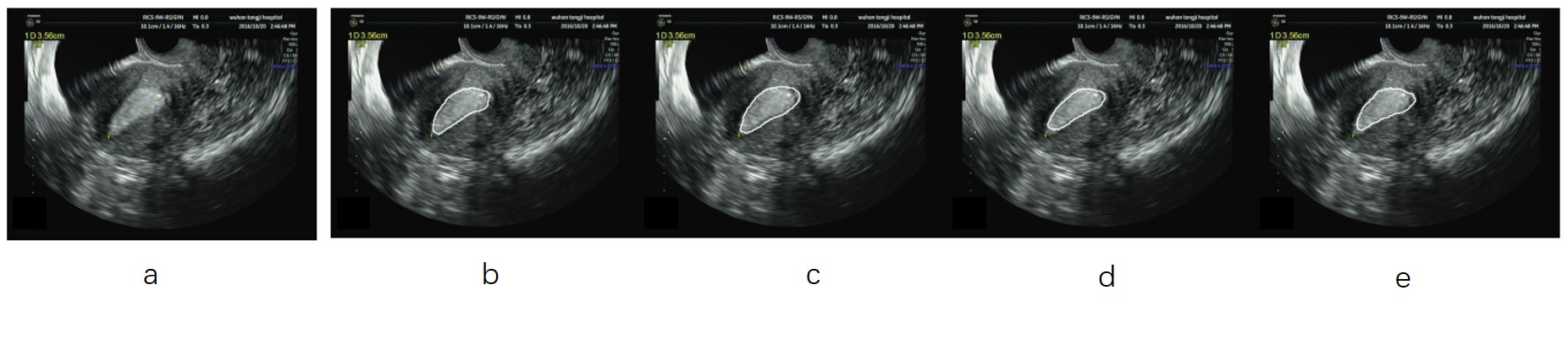

Supplement: Supplementary file 3 [file Image4.JPEG]

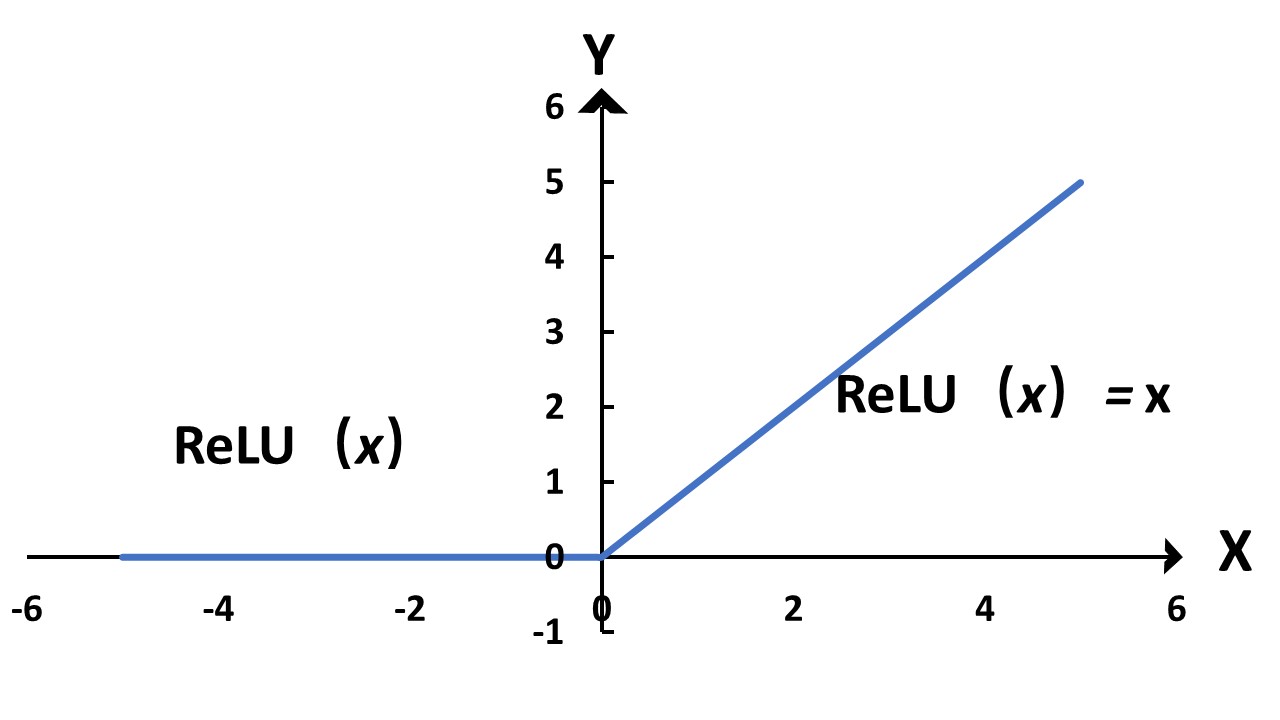

Supplement: Supplementary file 4 [file Image5.JPEG]

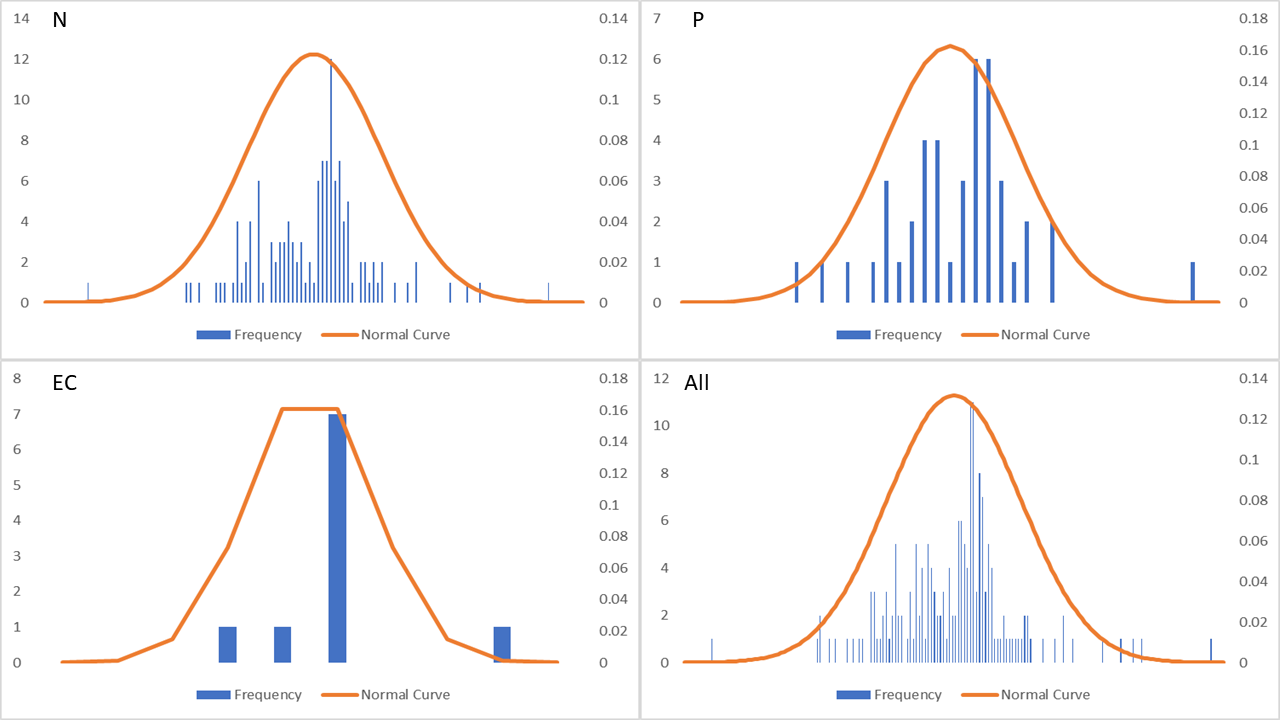

Supplement: Supplementary file 6 [file Image2.TIFF]

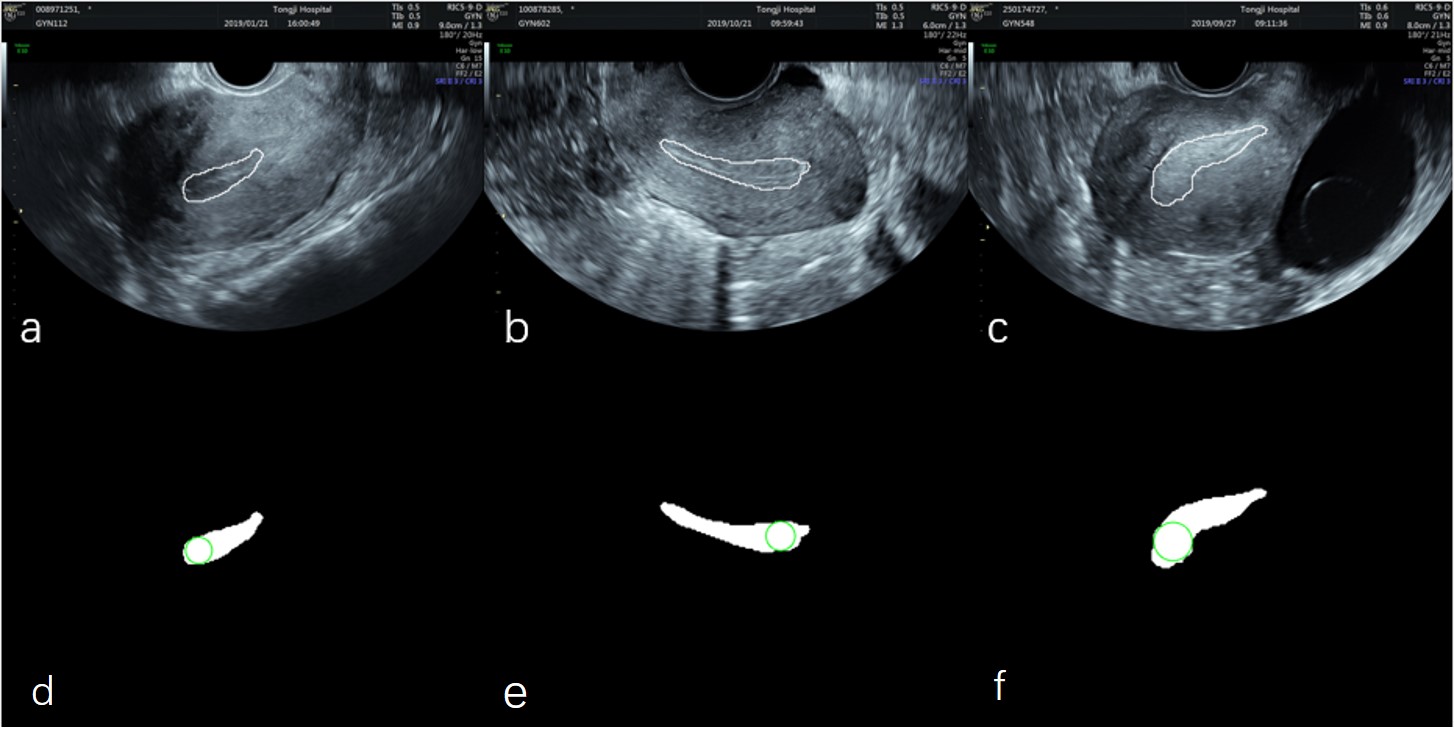

Supplement: Supplementary file 7 [file Image6.JPEG]
