# Supplementary material for: Automatic Measurement of Endometrial Thickness From Transvaginal Ultrasound Images
Source: Front Bioeng Biotechnol. 2022 Mar 29;10:853845. doi: 10.3389/fbioe.2022.853845 (PMC9001908; doi:10.3389/fbioe.2022.853845)
Supplement: Supplementary file 5 [file DataSheet1.PDF]

Supplementary Material

1 ACTIVATION FUNCTION

We use ReLU as the activation function, as follows.

$$\mathrm{ReL}\,U(x) = \begin{cases} x & \text{if } x > 0 \\ 0 & \text{if } x \leq 0 \end{cases} \tag{S1}$$

ReLU's curve is shown in Figure S1.

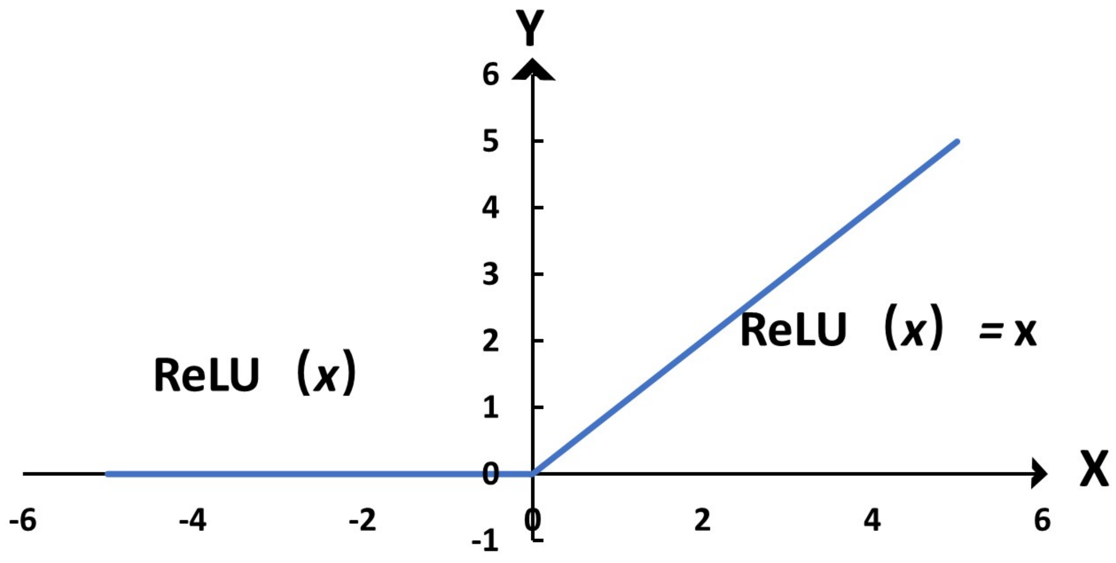

Figure S1. ReLU activation function Agarap (2018).

2 SEGMENTATION EXAMPLE

Figure S2(a) illustrates a uterine ultrasound image in the validation dataset. Figures. S2(b)-(e) show the segmentation results from ResNet50-SegNet, ResNet50-UNet, Vgg16-SegNet, and Vgg16-UNet, respectively. The corresponding Dice coefficients were 0.9376, 0.9164, 0.8750, and 0.9096, respectively. Through validation using 1059 image from 71 cases, the average Dice coefficients were 0.81, 0.72, 0.69, and 0.59 for the four segmentation models, respectively.

3 FLOWCHART OF THE PROPOSED METHOD

Figure S3 illustrates the flowchart of our two-step method. The original image is input to the segmentation model (SegNet-ResNet50) to obtain a mask image. Through combining the original image and the mask image, we get a segmentation result with a mask on the original image. In addition, a maximum inscribed circle is searched to approximate the boundary of the endometrium. The diameter of this inscribed circle is the thickness of the endometrium. We also extract the pixel spacing information in the header of DICOM file to calculate the actual diameter (in millimeters) of the inscribed circle image (indicated by the number

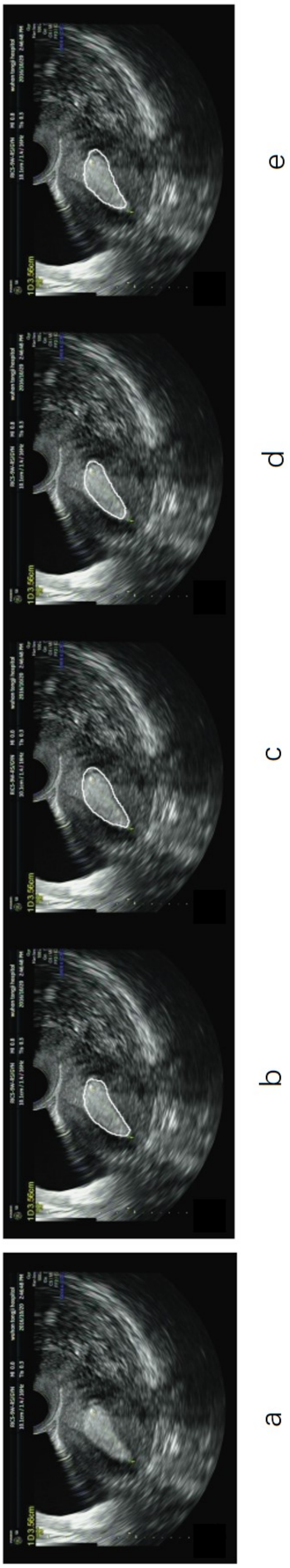

Figure S2. Segmentation results of the same image using different models. (a) uterine ultrasound image example from the validation dataset. (b)-(e) segmentation results from ResNet50-SegNet, ResNet50-UNet, Vgg16-SegNet, and Vgg16-UNet, respectively.

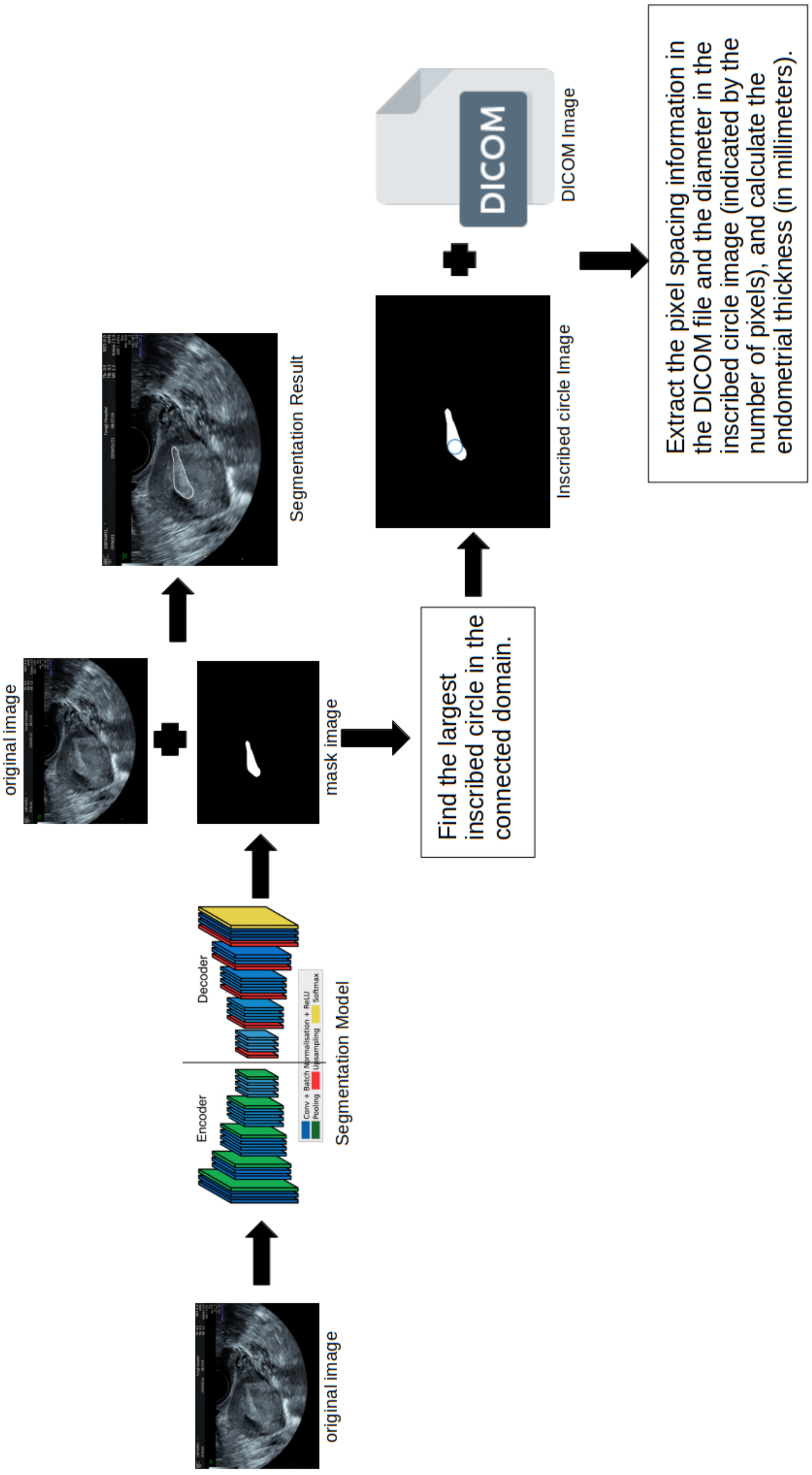

Figure S3. Flowchart of the proposed method for automated endometrial thickness measurement (modified from Badrinarayanan et al. (2015))

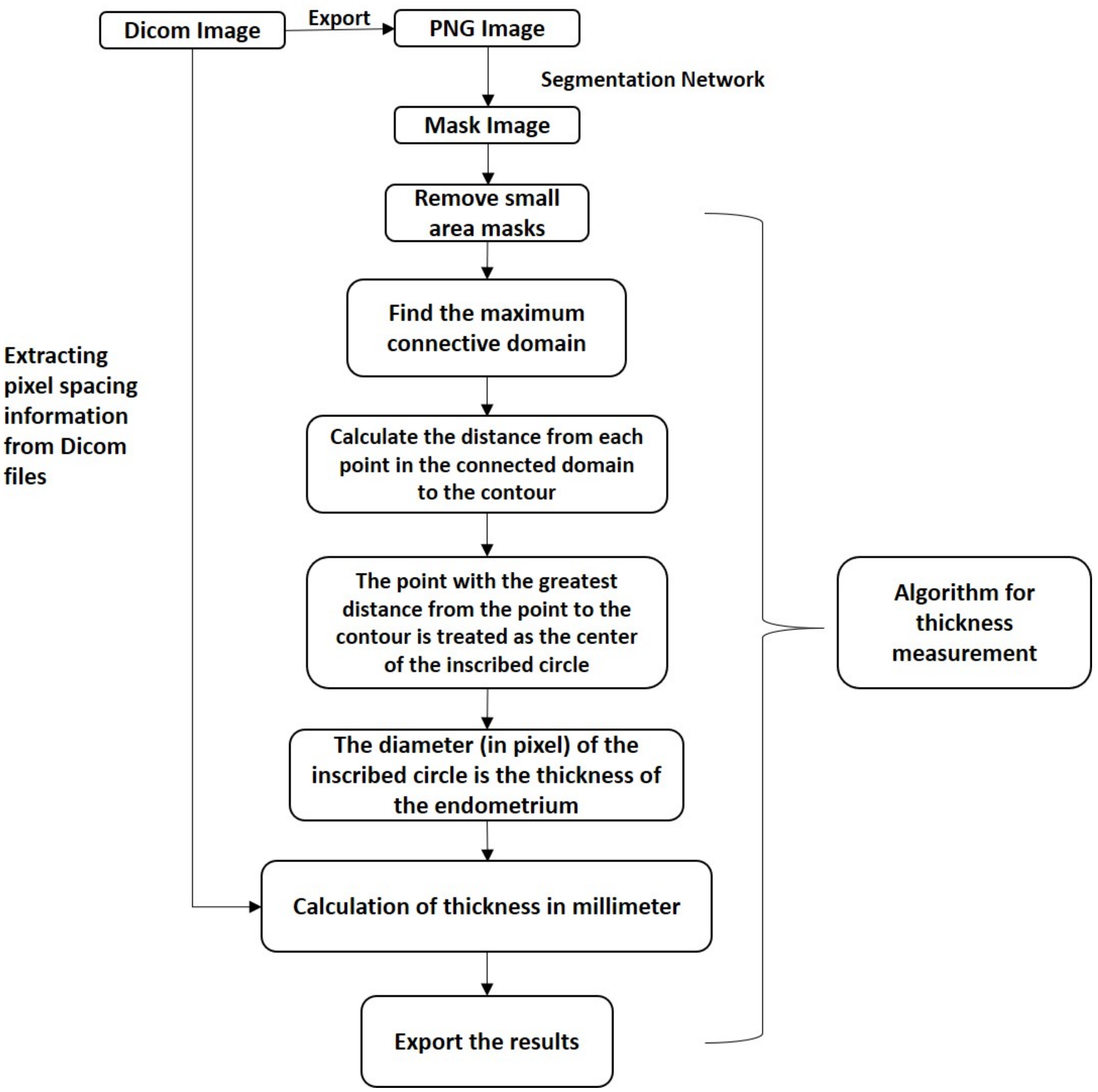

Figure S4. Flowchart of the maximum inscribed circle search method.

of pixels). Figure S4 illustrates the flowchart of the maximum inscribed circle searching method for ET measurement. Figure S5 shows the results of the largest inscribed circle found by the proposed method. Figures S5(a)-(c) is the original images with the boundaries of endometrium, and Figures S5(d)-(f) the segmentation ground-truth with the largest inscribed circle.

4 NORMAL DISTRIBUTION

Figure S6 shows the distributions of ET errors estimated from images of normal cases and those with endometrial diseases. The t-test analyses indicate no significant difference among ET errors estimated from normal cases (N) and those with endometrial polyps (P) or cancer (EC).

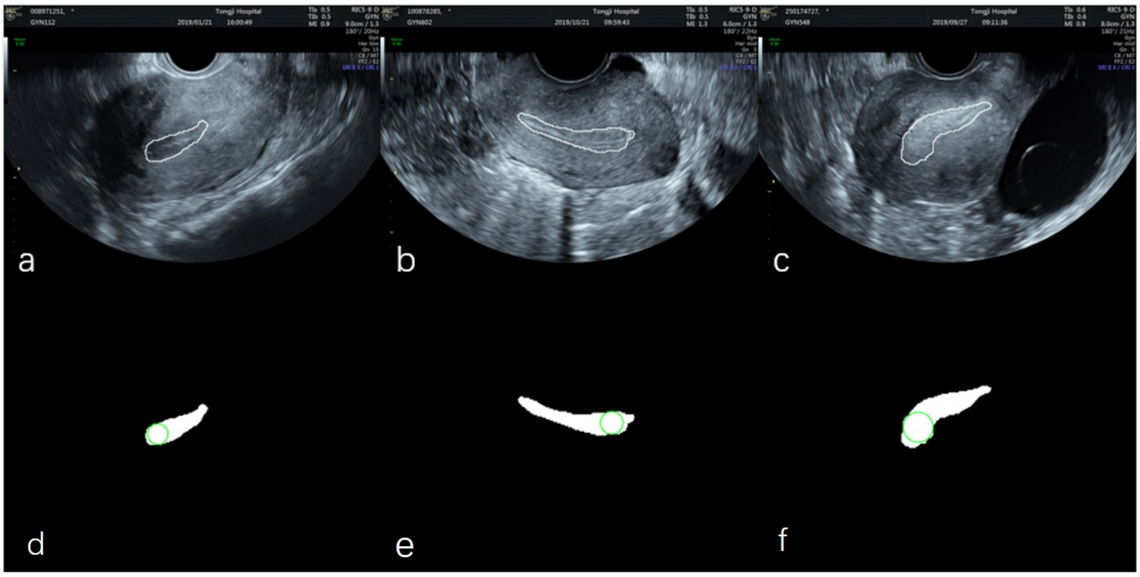

Figure S5. ET measurement results of using the largest inscribed circle searching method. (a)-(c) show image samples; (d)-(f) show the corresponding largest inscribed circles in the segmented endometrial masks.

REFERENCES

Agarap, A. F. (2018). Deep learning using rectified linear units (relu). *arXiv preprint arXiv:1803.08375* https://arxiv.org/abs/1803.08375

Badrinarayanan, V., Handa, A., and Cipolla, R. (2015). Segnet: A deep convolutional encoder-decoder architecture for robust semantic pixel-wise labelling. *arXiv preprint arXiv:1505.07293* https://arxiv.org/abs/1505.07293

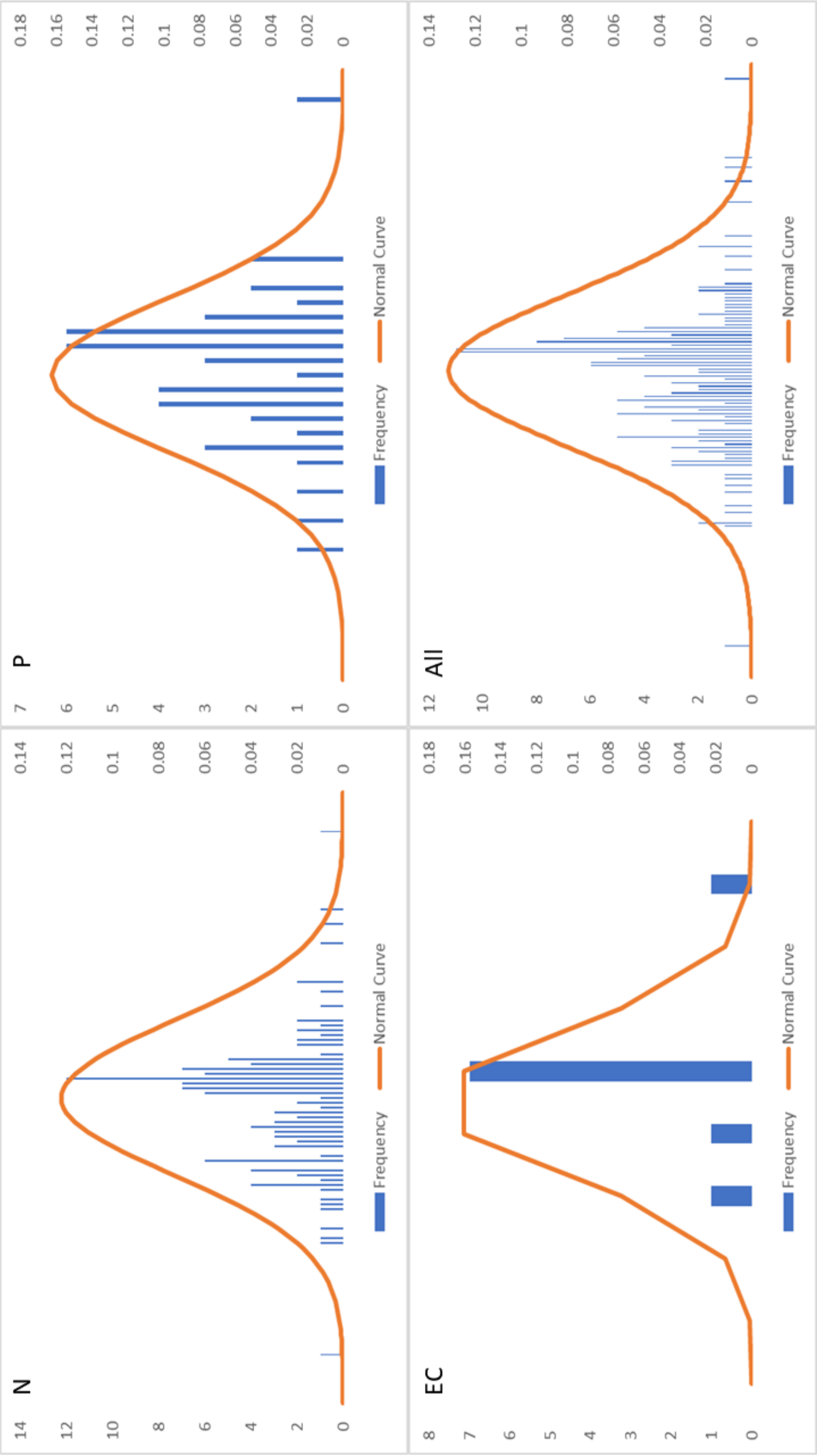

Figure S6. Distributions of ET errors estimated from normalN) caes, those with endometrial polyp (P), endometrial cancer (EC), and all cases
